# Do David and Goliath Play the Same Game? Explanation of the Abundance of Rare and Frequent Invasive Alien Plants in Urban Woodlands in Warsaw, Poland

**DOI:** 10.1371/journal.pone.0168365

**Published:** 2016-12-16

**Authors:** Artur Obidziński, Piotr Mędrzycki, Ewa Kołaczkowska, Wojciech Ciurzycki, Katarzyna Marciszewska

**Affiliations:** 1 Department of Forest Botany, Faculty of Forestry, Warsaw University of Life Sciences, Warsaw, Poland; 2 Laboratory of Applied Plant Ecology, Faculty of Ecology, University of Ecology and Management, Warsaw, Poland; 3 Department of Geoecology and Climatology, Institute of Geography and Spatial Organization, Polish Academy of Sciences, Warsaw, Poland; Chinese Academy of Forestry, CHINA

## Abstract

Invasive Alien Plants occur in numbers differing by orders of magnitude at subsequent invasion stages. Effective sampling and quantifying niches of rare invasive plants are quite problematic. The aim of this paper is an estimation of the influence of invasive plants frequency on the explanation of their local abundance. We attempted to achieve it through: (1) assessment of occurrence of self-regenerating invasive plants in urban woodlands, (2) comparison of Random Forest modelling results for frequent and rare species. We hypothesized that the abundance of frequent species would be explained better than that of rare ones and that both rare and frequent species share a common hierarchy of the most important determinants. We found 15 taxa in almost two thirds of 1040 plots with a total number of 1068 occurrences. There were recorded 6 taxa of high frequency–*Prunus serotina*, *Quercus rubra*, *Acer negundo*, *Robinia pseudoacacia*, *Impatiens parviflora* and *Solidago* spp.–and 9 taxa of low frequency: *Acer saccharinum*, *Amelanchier spicata*, *Cornus* spp., *Fraxinus* spp., *Parthenocissus* spp., *Syringa vulgaris*, *Echinocystis lobata*, *Helianthus tuberosus*, *Reynoutria* spp. Random Forest’s models’ quality grows with the number of occurrences of frequent taxa but not of the rare ones. Both frequent and rare taxa share a similar hierarchy of predictors’ importance: Land use > Tree stand > Seed source and, for frequent taxa, Forest properties as well. We conclude that there is an ‘explanation jump’ at higher species frequencies, but rare species are surprisingly similar to frequent ones in their determinant’s hierarchy, with differences conforming with their respective stages of invasion.

## Introduction

### Problems of species frequency

Research on the invasion process is heavily impacted by the fact that in different stages–'transport', 'introduction', 'establishment', and 'spread' [1]–self-regenerating invasive alien plants (IAP) occur in numbers differing by many orders of magnitude. Not only does this pose problems in effectively sampling less frequent species, but it also constitutes a serious challenge in quantifying their niche and the importance of the barriers that slow down the course of an invasion.

During the initial stages of their invasion, IAPs occur rarely in the neighbourhood of transport facilities or near cultivation places. In later phases, their populations usually grow and disperse into new places and habitats. The success of a species in that later phase is seen in the passage from surviving as individuals to self-sustaining abundant populations. This transition may be accelerated or slowed down by the number of biotic or abiotic interactions [2]. Their further spread may also be restricted by low availability of suitable habitats, e.g. *Vaccinium mactocarpon* on peatlands.

**Fig 1:**
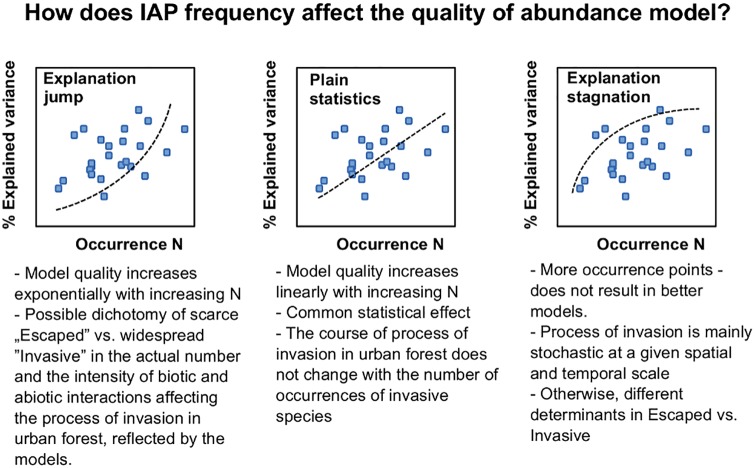
Model quality and the frequency of IAP species.

The greater the species frequency, the higher would be the importance of niche relationships. But still an invasion process occurs in a quite stochastic way, benefiting from unique or hardly quantifiable stochastic disturbances [3]. When their impact on a given invasion case prevails, species differentiation and competitive abilities should not be very important. (Fig 1).

When the number of actual interactions increases with growing frequency, the explained part of the variation should rise and have less random variability, while in the case of high stochasticity of the process, the explanation of more frequent species should not be much better than rarer ones and the values for both species group should be equally variable.

These biological patterns may be obscured by purely statistical effects, i.e. decreasing power of the test when sample size decreases or by the results of data imbalance, i.e. the prevalence of absences over presences for rare species. Some statistical treatments, e.g. oversampling of minority classes, are advised as a solution to unbalanced data analysis [4].

Finally, the quality of the models for rare and frequent species also depends on spatial scales in which the occurrence of frequent and rare IAP species were described and analysed. Choosing the right scale compatible with response and predictor data should enable proper matching of the possible biological cause and effect that interplay during an invasion case [5,6]. A serious mismatch may be responsible for the model’s irrelevance.

The identification of determinants of a given IAP success is a *sine qua non* condition for the design of prevention or control measures. This is the stage at which target-specific preventive and early-detection measures are able to act quickly and efficiently, for which the predictive capabilities of statistical models are badly needed [7]. Therefore, also from a practical point of view, answering the question of the modellability of early phases of invasion is important.

### Urban woodlands as a test bed of invasions

Urban woodlands are particularly suitable for testing this issue. Forest complexes in urban landscapes form an archipelago of islands of different sizes and shapes, with specific historical and neighbourhood properties. This enables fairly easy quantification of both IAPs and invaded forest environment traits used in statistical modelling. Urban woodland, e.g. in Berlin, are more invaded [8] than natural forests [9]. Moreover, the intensity of invasions varies widely between different urban forest complexes, which is an opportunity to study determinants of forest habitat invasibility.

### Main factors influencing spread of invasive alien plants

There are several factors determining the pace of IAP establishment and spread. The ones considered most important are as follows: (**a) *Seed source*:** The rate of forest invasion inside urban settlements may result from variability in distances to and the abundances of seed sources. **(b) *Facilitation*:** Previously established IAP may promote the establishment of populations of other IAPs. Such a process is considered to be a facilitation [10] or even mutualism [11]. **(c) *Tree stand and habitat properties*:** The growth of juvenile forms of woody species depends much on the canopy species’ composition and the canopy structure [12], which in turn is a function of the forest dynamic phase [13]. Thus undisturbed late-successional fragments are rarely invaded by IAPs, while early-successional or frequently disturbed forest communities are easily invaded [14]. Soil fertility and moisture also have significance for efficiency of IAP settlement [15]. **(d) *Land use*:** The neighbouring land use has been mentioned as a crucial determinant of urban flora composition in general [16]. Humans penetrating forest space perturb the soil and the plant cover, which may foster establishment of IAPs [17]. Indirectly, they may generate the urban heat island effect [18]. Nitrogen and sulphur oxides released from home heating contaminate soil all over the cities [19], whereas chloride salts and heavy-metal pollutions disperse along urban roads [20]. In effect, many thermophilous, nitrophilic, salt- and heavy-metal-tolerant plants are favoured in urban areas [21–23].

### Quantitative studies

To date, some studies have assessed the importance of habitat characteristics, tree stand properties, the effect of facilitation between alien species, forest patch traits, or surrounding land use on IAPs’ dispersal (Table 1). The biggest limitation of many of those studies is their statistical methods. Some of them use single variable statistics that do not allow for joint assessment of the importance of more than one trait. Others use parametric regression statistics (GLM), which are sensitive to skewed predictor dispersal with outliers.

**Table 1:** Quantitative studies on IAPs’ distribution.

Study	Analysed forest traits	Statistical method	Number of species	Number of repetitions	Model quality R^2^
[26]	fragment area, canopy closure within the fragment, fragment age, and fragment heterogeneity, residence time in Austria	ANCOVA, Canonical Correspondence Analysis	62	44	0.3 to 0.35
[27]	adjacent habitat diversity (presence/absence of: large roads, railways, crop fields, built-up areas); forest patch size, species ratio alien/native	Single Poisson Generalised Linear Models	na	15	0.64
[28]	minimal distance to settlements, forest types, forest habitat type, adjacent habitat diversity, the presence/absence of the following five habitat types within the buffer distance: large roads, railways, crop fields, built-up areas, and dry grasslands	Linear Mixed Models and Generalised Linear Mixed Models	159	30	na
[29]	settlement size, planting age, number of people, cultivated species, non-cultivated species	Generalised Linear Models	85	36	na
[30]	invasibility of riparian forest, relative alien cover (%), watershed characteristics: area [km^**2**^], impervious surface (%), stream hydrology; canopy gap (%), species richness, Shannon diversity, nutrient content, soil pH	Single Linear Regression	na	na	0.0 to 0.7 for single trait
[31]	water quality, river bank stability	Bivariate correlations	51	18	0.06 for single trait
[32]	climate variables, factors related to nutrients, land cover	Species Distribution Models (Random Forest)	11	1329	(AUC > 0.7)
[33]	climate variables, land cover, nutrient properties of parent rock	Species Distribution Models (Random Forest)	34	1393	(AUC > 0.7)

Only a few studies have taken advantage of less sensitive to the data parameters data-mining algorithms such as Random Forest [24] or gradient boosted trees [25], which enable a joint analysis that includes all available, consistent data. Not all of the analyses used feature-selection options, and where used—for example, AIC criterion in GLM or GLMM models—they were aimed at finding the simplest models with the best fit to the data. This may have resulted in the dropping of some weaker but still biologically relevant variables from the models. (Table 1)

Most regression-type models had their quality expressed in a universal unit, e.g. R^2^ or its equivalents (pseudo R-squared), which usually varied from 0.3 to 0.6, with more variation in single bivariate regression models than in multiple variables models. An unknown portion of this parameter (R^2^) could have resulted from the differences in the power of the test, sample size, data imbalance, or a biasing estimation of biological effect.

Because of these limitations, quantitative meta-analysis of already published materials is hardly possible. New evidence coming from empirical studies would be of help in estimating the modellability of rare IAP species in comparison with more frequent species. For that purpose, most recent, insensitive to the data parameters statistical methods should be used, and the frequent and rare species' response to as many predictors as possible should be assessed, using the same set of sampling areas within an ecologically coherent space and time. Cross-species and cross-feature group comparisons of relative importance should be possible, together with an estimation of effect of data imbalance on the variance explained by the model. A research area should be large and variable enough for making a sufficient number of feature combinations possible. Ideally, it would require an area with both newly created forests and the remnants of old woodlands of variable size and shape and surrounded by various land use types.

## Aim of the study

The first aim of this study was to perform a representative assessment of the abundance of IAPs in Warsaw’s municipal urban woodlands and an estimation of the hierarchy of importance of variables for all chosen species using robust model algorithms with a coherent dataset for seed source, inter-IAP facilitation, soil, tree stand features, forest history and properties, and the type of surrounding land use as predictors. We then aimed at answering the following questions for rare and frequent IAP species: **(a)** How does the frequency of the IAP species affect the explanatory ability of the models? **(b)** Do single variables and variable groups differ in the importance and significance between frequent and rare species? **(c)** Do both groups (i.e., frequent and rare) behave so similarly that they represent the same biological process (of colonization), so that they can be considered as one statistical population and be calculated together in the same model?

Since single variables and groups of similar variables included in this study cover a broad spectrum of potential determinants, we expected that the explained part of the variation of local IAP abundance would be at least as much as in models published so far (i.e. ≥ 30%).

Since better quality of frequent species models would result from their better adjustment to their habitats (in large part caused by their longer time of colonization and broader spread of seed rain), and the lower quality of rare species models would result from higher stochasticity of their occurrences (caused by the scarce, habitat-independent anthropogenic localization of their seed sources) we also expected that: **(a)** more frequent species would be more limited or fostered by the biotic and abiotic relationships, so that abundance of frequent species would be explained in larger part than rare ones; **(b)** frequent species would have less variability in the amount of the explained variance than the rare ones; and **(c)** the hierarchy of importance would be distinctively different in rare and frequent species. We also expected that the presence of seed sources and habitat conditions would be the most important among all the predictors.

## Materials and Methods

### Study area

Warsaw, the capital of Poland, currently inhabited by 1,711,324 citizens [34], lies between coordinates 52°05’48” to 52°22’11” N and 020°51’04” to 021°16’26” E, on the River Vistula at elevation 77–116 m a.s.l. It is located in the temperate continental climate zone [35]. The average annual temperature is 8°C; the average in the coldest month (January) is -3°C and 18°C in the warmest month (July) [36].

Woodlands in Warsaw cover 14% (72.6 km^2^) of the total city area [37]. The term “Urban woodlands—Warsaw” refers to the woodlands of municipal ownership. It is 38% of total woodland areas, while the private forests comprise 44%, and state forests 18% [37].

**Fig 2:**
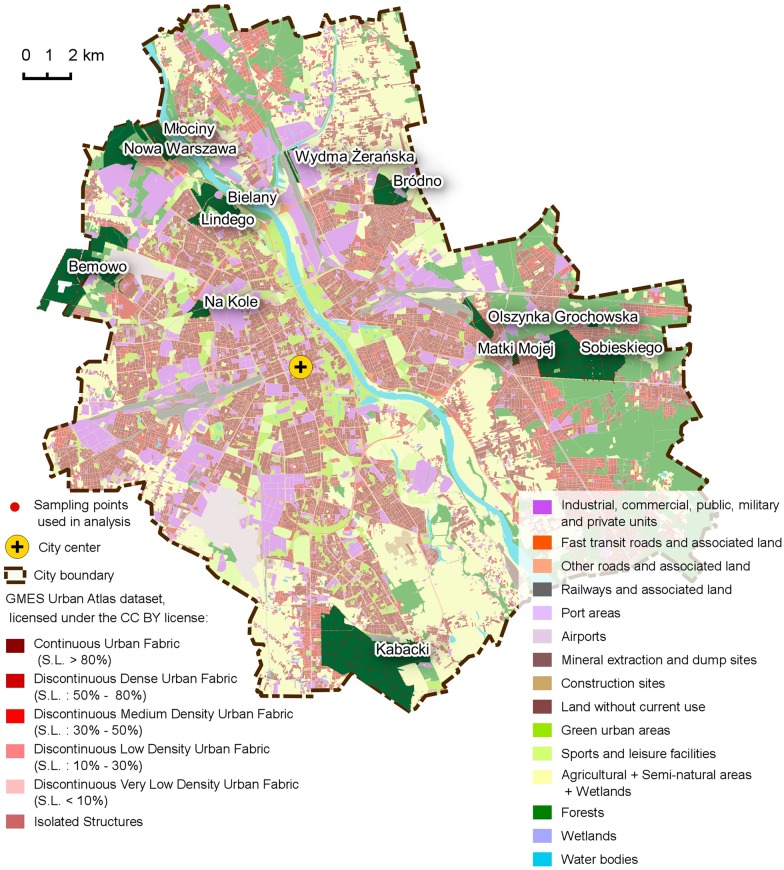
Location of studied forests and SP included in analysis. In the background—the Global Monitoring for Environment and Security Urban Atlas dataset, licensed under the CC BY license [41].

The western, major part of the city is situated mainly on the old glacial plain with rich, postglacial clay soils. The eastern part is located mainly in the river valley, with floodplains and sand dune terraces covered by sandy soils accompanied locally by marsh and peat deposits [38,39]. Urban municipal woodlands in Warsaw are located mainly on the podzol and lessive soils, less frequently alluvial soils, peats and mucks. According to the phytosociological classification the most common vegetation types of the woodlands are mixed pine-oak forest (*Querco roboris-Pinetum* J.Mat. 1988), oak-hornbeam forest (*Tilio-Carpinetum* Tracz. 1962) and locally fresh pine forest (*Leucobryo-Pinetum* W.Mat. 1973) [40]. Most patches are dominated by the Scots pine *Pinus sylvestris* L., silver birch *Betula pendula* Roth., or pedunculate oak *Quercus robur* L., but 18 other native tree species are reported from the woodlands according to forest management plans. The main function of the woodlands is sustaining the environment and providing a recreation facility for the city’s inhabitants [37]. The size of the forests varies from 0.1 to 1000 ha (Fig 2, S1 Table).

### Urban woodlands in Warsaw as a model system

Urban woodlands in Warsaw are well-suited to be such a model system. Of the total number of 1279 plant species recorded in Warsaw, alien species constitute almost 40% and 15% are established [21]. Urban woodlands in Warsaw have a wide distribution of size, shape and history. There are ancient woodland areas, forests regenerating after clearings and secondary forests that emerged from 20th-Century forest plantations. There are also documented IAP plantings, carried out in the years 1960–1980 (P. Prądzyński, personal communication, 2013). IAP species occur both alone and in tree stands, which makes them good areas for testing the effect of inter-IAP facilitation. Land use of surrounding forest patches varies from remnant agricultural fields and extensive residential areas, through fast transit roads, railways and airports, to dense housing estates and industrial areas. For analysed forests, many sources of standardized data are available. Land use was recently described during the Global Monitoring for Environment and Security Urban Atlas Europe-wide program of urban mapping by the European Environmental Agency [41]. Data on the tree stand, soil, and seed sources are available from the forest management plans.

The potential bias of the results obtained in the Warsaw municipal urban woodlands is the uneven distribution of their distance to the centre of the city. However, as Warsaw’s spatial structure is rather typical for many old, European cities [42] also Warsaw’s woodlands resemble other European cities woodlands. Finally, there are no recent, comprehensive studies of alien plant invasions in Warsaw forests so far, but local, unpublished studies confirm the presence of IAPs in many forests (e.g. [43–45]).

### Research procedure

**Fig 3:**
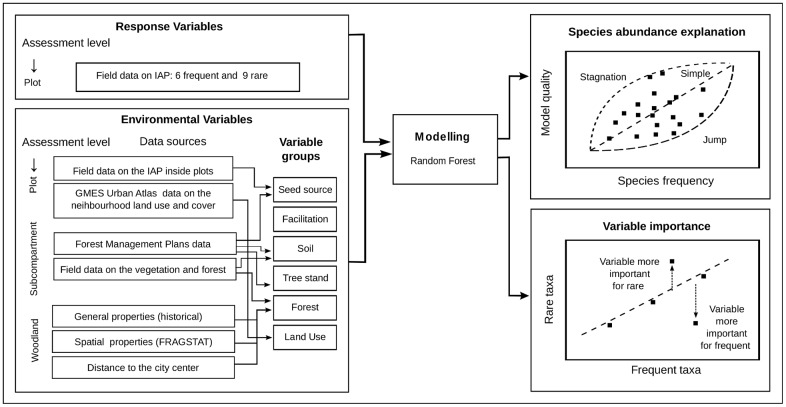
Schematic diagram of the calculation procedure.

The procedure included seven steps: (a) an algorithm choice, (b) IAP species choice for census, (c) field data collection, (d) predictors collection, (e) data preprocessing and statistical modelling with assessment of the model quality, (f) computing partial dependence plots and automatic selection of significantly relevant features, (g) comparison of the reaction of rare and frequent species to the environmental factors. (Fig 3)

### Algorithm choice

We have chosen the Random Forest (RF) algorithm [46,47] for the analyses for following reasons. The analysis of heterogenic data obtained from the studies of natural systems (“natural experiments”), being under little or no control of the researcher, require the use of an algorithm robust enough to cope with non-normal distributions of variables and capable of detecting nonlinear data relationships [48]. The algorithm should also be able to include a large number of variables in the analysis, to assess their importance, and to select features significantly related to the response variables. Its implementation in an open statistical environment like the R Statistical Environment [49] would enhance replicability of analyses. All those requirements are met by the Random Forest (RF) algorithm. It may perform analyses with up to thousands of variables, and is insensitive to the distribution and multicollinearity of independent variables [50]. It also has its reference implementation in the R randomForest package [51].

The advantage of Random Forest over similar algorithms is an additional “wrapper-algorithm”, implemented in the R Boruta 3.0 package [52]. It is an implementation of “an all-relevant feature selection wrapper algorithm. It finds relevant features by comparing original attributes’ importance with importance achievable at random, estimated using their permuted copies” [53]. The Boruta wrapper performs 150 Random Forest iterations and then performs a significance test of differences of means. Features whose importance is not significantly different from the average importance of their randomized copy are rejected. Parameters that differ significantly are labelled “Confirmed”. Those on the verge of significance are labelled “Tentative” and may be resolved through additional procedures, if necessary.

### IAP species choice

As the field census had to deliver reliable and accurate data on the distribution and abundance of the most important IAPs across all studied forests in a short period of time, we decided to limit the set of recorded species to those which: (1) were listed among the most invasive species in Poland and Europe [54,55–57], (2) were already found self-regenerating in the city, and (3) whose field identification were undoubted. The list was further supplemented by a few species that are less aggressive, but common in Warsaw ([21], personal observations). Congeneric species pairs–*Cornus alba* and *C*. *sericea*; *Fraxinus ornus* and *F*. *pennsylvanica*; *Parthenocissus inserta* and *P*. *quinquefolia*; *Reynoutria japonica* and *R*. *sachalinensis*; *Solidago canadensis* and *S*. *gigantea*–were assessed jointly in order to avoid problems with determination by field observers. Further in the text, species of each pair are described with the abbreviation “spp.”. Finally, the set of censused species contained 10 woody and 6 herbaceous species (S2 Table).

### Field data collection

**Fig 4:**
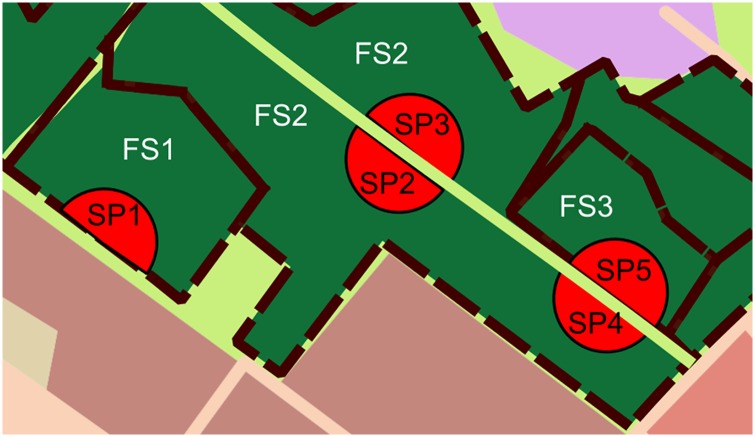
The scheme of sampling plots location. SP = sampling plot, FS = forest subcompartment. SP1 is located near the outer border of the forest in the forest subcompartment FS1. SP2 and SP3 are located on both sides of a forest path, but in the same forest subcompartment FS2. SP4 and SP5 are located on both sides of the path, but in separate subcompartments FS2 and FS3.

In order to obtain consistent field data from 12 forest complexes during a short time, we decided to use sampling plots (SP), located along the borders of each forest and main forest roads (Fig 3). SPs were half-circles with a radius of ca. 25 m, located independently on both sides of the forest road, or only on one side, when applied to the forest border (Fig 4). The distance set between plots was 250 m. As complete forest management plan data were not available for private ownership woodlands, only municipal urban woodlands were covered during the field census. In forests where ownership is a mosaic the sampling was therefore more dispersed.

In each and every SP an abundance of each woody IAP was assessed separately in each of the forest layers (A = tree stand, height more than 5 m; B = shrub layer, 0.5–5 meters; C = herb layer, less than 0.5 m), while herb species were assessed only in the C layer. Abundance was assessed with a rank scale based on the species cover, composed of 4 categories: “0” = absence of the species, “1” = “single individuals or species covers <10%”, “2” = “species common, covers 10–50%”, “3” = “species abundant (covers >50%)”. For each SP, documentation was made with an 8 MP digital camera and geographical position was registered with Garmin eTrex H GPS units. Data collection was performed in August and September 2012.

### Response variables

The response variables were: the abundance in C layer for herbaceous IAP species and sum of abundance in layers B and C for woody IAP species. Sites with “0” abundance (absence) were includes in the modelling. Woody IAPs’ abundance in the canopy layer was set to be one of the predictors. The authors are aware that this may have resulted in a more conservative assessment of the scale of the IAPs’ invasion. However, in this way we avoided the bias resulting from counting the purportedly planted individuals into the abundance of spontaneous colonization. Therefore, the modelling result with B+C layers’ abundance as a response variable may be interpreted as probability of self-regeneration of IAP—a crucial phenomenon during initial stages of biological invasion [1].

### Predictors

For predictors we adopted data from forest management plans, data from previous land cover mapping [41], data published in the literature, as well as our own field assessment. Some of the variables were recomputed using QGIS 2.2 software [58]. The full list of predictors can be found in S3 Table.

#### Habitat conditions and tree stand predictors

Data on habitat conditions in the forest compartments in which SPs were located, imported from forest management plans, included: the type of habitat according to Forest Service classification, the soil cover type, the soil cover shade tolerance index, the trophic index of the soil, the index of wetness of the soil. From the same source we extracted predictors describing tree stands with SPs located in them including: forest compartment conservation status, the present vegetation type based on the national classification by [59] the naturalness of the stand, tree species with the highest abundance in the canopy, the index of stand growth quality for the compartment, the age of the present stand, and the relative density of the canopy layer. In this predictor group we added a few more variables such as the canopy shade index, which was calculated as a mean of the shading coefficients [60] for all species locally present in the canopy, weighted by each species’ cover coefficient in the canopy taken from the forest management plans. The distance from the SP to the forest border was measured using the digital forest maps in GIS; vegetation deformation level is specified by [61], and vegetation degeneration level assessed personally in the field.

#### Forest properties

Among predictors describing the properties of whole forests in which SPs were located, there were: the historic type of the forest, the distance from the forest centroid to the administrative city’s centre (see Fig 2), the relative abundance of urban infrastructure in the forest, such as roads, paths, parking areas, banks, underground and aboveground pipes and cables, etc., and the naturalness of the herb layer, assessed in the field. In order to assess spatial characteristics of forests, predictors describing size and shape of the forests were calculated. The total forest area and perimeter of the forest, including any internal holes, were measured in GIS on digital vector SHP maps from the Forest Service. In addition, shape characteristics were computed on the rasterized digital maps using the PatchStat function from the SDMTools package for R [62], which included the forest core area index, meaning: “core area as a percentage of patch area”, the shape complexity index, meaning the “sum of each patch’s perimeter divided by the square root of patch area”. Other shape indexes available in this function were omitted because of their high correlation rate with the two mentioned. To measure the distance from the SP to the forest border the Urban Atlas of Warsaw [41] was used. It describes the most recent available state of land use / land cover of the Warsaw agglomeration in the years 2005–2007, prepared during the Global Monitoring for Environment and Security program by the European Environmental Agency (EEA), published under the CC-BY license and freely available on the Agency site (http://www.eea.europa.eu/data-and-maps/data/urban-atlas). The distances were calculated in GIS.

#### Facilitation predictors and seed source

The first group of facilitation predictors includes the presence of the same species in the tree layer in the same SP; the second, the distances to the nearest compartments with the same species present in a canopy. The data on the presence of all other species in the canopy, shrub or herb layer separately, were included in analysis as potential facilitation predictors. Data on the seed sources were available for woody species only.

#### Land use predictors

Urban Atlas was the source of data of surrounding land use. All predictors were assessed as a mean minimal distance from the SP to the three nearest border points of each Urban Atlas unit type. Border points were randomly located every 10 m along the borders of the each Urban Atlas patch. This group of predictors includes the distances to the nearest patch of Agricultural+Semi-natural areas+Wetlands, Airports, Construction sites, Continuous Urban Fabric (SL > 80%), Discontinuous Dense Urban Fabric (SL: 50%-80%), Discontinuous Medium Density Urban Fabric (SL: 30%-50%), Discontinuous Low Density Urban Fabric (SL: 10%-30%), Discontinuous Very Low Density Urban Fabric (SL: 10%-30%), Mineral extraction and dump sites, Industrial+Commercial+Public+Military units, Railways and associated land, River ports, Fast transit roads and associated land, Other roads and associated land, Sports and leisure facilities, Land without current use, and Water bodies.

After compilation the dataset had 75 variables and 1233 rows.

### Data preprocessing

During data preprocessing, the rows with some missing data were excluded. Then, the variables with near-zero variance predictors were removed. The identification of near-zero variance predictors was performed using the nearZeroVar() function from the R package caret [63] including freqRatio—the ratio of frequencies for the most common value over the second most common value—and percentUnique, the percentage of unique data points out of the total number of data points, with default values. The excluded 19 variables with near-zero variance represented mainly rare IAP species abundance variables. However, in Random Forest models, the abundances of low frequency taxa were used as response variables. After the preprocessing the dataset had 56 variables and 1040 rows.

### Models computation

Single Random Forest models were computed using the R package randomForest ver. 4.6.7 with all default values except the n-tree parameter, which was set to twice the default value (1000) in order to increase the stability of results. Models were computed using the option regression = TRUE, in which the parameter “pseudo R-squared” is computed, using Mean Square Error with the formula: pseudo R-squared = 1 –MSE / Var(Y) [64]. The pseudo R-squared parameter is an equivalent to R^2^ in regression models and approaches 100% when the variance explained by the model approaches the empirical variance of the response variable.

Negative values occur when the Mean Square Error surpasses the variance of dependence values. In order to assess the shape of the relationship between model quality expressed by pseudo R-squared and the number of presence cases for each species, the normality of both variables was tested in PAST statistical software, v. 2.16, using 4 normality tests: Shapiro-Wilk, Jarque-Bera JB, Chi^2^, and Anderson-Darling [65]. There was no departure from normality in one variable (model quality) and there was little ambiguity in the second variable (the number of presences) when it was log-transformed (2 of 4 tests were marginally significant). The Spearman’s rho correlation coefficient was calculated as it does not require normality assumptions. It was computed for all models and for subgroups including models for single species as a biologically coherent group. In order to elucidate the shape of the relationship, the LOWESS (LOcally WEighted Scatterplot Smoothing) algorithm from the PAST software was used, which produces a smoothed line [66].

In order to compare the importance of variables and variable groups, RF IncNodePurity importance was computed. As there are no built-in functions in the package randomForest that allow for computing the total importance of variable classes, we decided to test which measure of the overall importance would be a better estimate of the importance of variable groups. In order to do the test, we computed 4 RF models with settings identical to those in the main analysis, with abundance of *Impatiens parviflora* as a response variable and 4 specially prepared predictor datasets. The first consisted of original data with 56 variables, the second with all variables except the response repeated 2 times, the third with variables repeated 4 times, and the fourth 8 times. The real explanatory capabilities of these predictors was not affected by the repetitions. The measure that remained stable could have been adopted as a good estimator of the total importance of variables. The average value of mean IncNodePurity importance was 2.96±SD = 2.14 and decreased roughly twice each time the number of predictors was duplicated, by 4 times if there were 4 copies, and 8 times if there were 8 copies, in comparison with the original data: it was 5.80 for the first dataset, 3.32 for the second one, 1.79 for the third one, and 0.93 for the fourth. The average of all importance sums for the models was 316.33±SD = 2.12, and it amounted to 313.60 for the first dataset, 315.74 for the second one, 317.69 for the third one, and 318.29 for the fourth. Given the random character of the RF analysis, the sum remained fairly stable across models with different amounts of predictors with exactly the same predictive power, so we chose it for a measure of summary importance of variable groups. The raw IncNodePurity was taken as a measure of single variable importance.

In order to find significantly important predictors for each species, feature selection was performed with the R package Boruta, v.3.0.0 [52]. Variables labelled as 'Confirmed’, with importance significantly greater (at p ≤ 0.01) than the importance of their randomized copies, were treated as significant predictors.

### Ethical considerations

The study did not involve disturbing protected species, and entering protected areas took place only through publicly available ways. Therefore, it was not covered by Polish law concerning ethical hearings requirements in research, and no permissions were sought from any ethics committee. I state clearly that no specific permissions were required for any locations and/or performed activities, as we walked only through publicly available roads or tracks and did not enter the forests nor collect any plants or their parts.

## Results

### Species distribution

There were overall 1068 IAP occurrences, found in two thirds (658 of 1040) of SPs (Table 2). Between 0 and 7 IAP species were found in each SP. There were 396 plots with one IAP, 167 plots with two IAPs, 60 plots with three, and 35 with four or more species. Mean abundance was 1.32 in all SPs, and 2.09 in SPs with any IAP. The most common IAP species in urban woodlands in Warsaw are *Impatiens parviflora* (found in 19.3% of SPs) and *Solidago* spp. (10.5%) among herbaceous IAPs, and *Prunus serotina* (18.7%), *Quercus rubra* (17.0%), *Acer negundo* (16.4%), and *Robinia pseudoacacia* (11.7%) among woody IAP.

**Table 2:** The occurrence and abundance of frequent and rare IAP taxa in municipal urban woodlands. Calculated on the dataset of 1040 Sampling Plots (SP).

Taxa	The number of forests colonized	The number of SPs with a given IAP presence	The share of SPs with a given IAP presence (%)	Mean of abundance of a given IAP in all SPs (%)	Mean of abundance of a given IAP in SPs where it is present (%)
Frequent species					
*Acer negundo*	12	171	16.4	0.28	1.71
*Impatiens parviflora*	10	201	19.3	0.25	1.31
*Prunus serotina*	10	194	18.7	0.25	1.36
*Quercus rubra*	9	177	17.0	0.28	1.62
*Robinia pseudoacacia*	12	122	11.7	0.27	2.31
*Solidago* spp.	11	109	10.5	0.15	1.45
Rare species					
*Acer saccharinum*	3	7	0.7	0.01	1
*Amelanchier spicata*	5	8	0.8	0.01	1
*Cornus* spp.	3	8	0.8	0.01	1.5
*Echinocystis lobata*	2	7	0.7	0.01	1.43
*Fraxinus* spp.	7	17	1.6	0.02	1
*Helianthus tuberosus*	6	12	1.2	0.02	1.75
*Parthenocissus* spp.	9	27	2.6	0.04	1.56
*Reynoutria* spp.	5	6	0.6	0.01	1.17
*Syringa vulgaris*	8	12	1.2	0.02	1.5

The mean abundance was the highest in *Acer negundo* and *Quercus rubra* (0.28) and *Robinia pseudoacacia* (0.27) followed by *Prunus serotina* and *Impatiens parviflora* (0.25). The six most common IAP species were found in almost all the forests. The only exceptions were *I*. *parviflora* and *R*. *pseudoacacia*, not found in one forest. Beside these six species, all but *Parthenocissus* spp., occurred in less than 2% of SPs. One species preliminarily enlisted for census, *Rudbeckia laciniata* L., was not found in any SP.

### Forest colonization by IAP

There was no municipal urban woodland in Warsaw free from IAPs. In each of them there were between 3 and 13 (average 9.3 ±SD = 3.3) IAPs. The average frequency of SPs with IAPs was 79%±SD = 19%, ranging from 44% to 100%. There were from 0 to 3 IAPs (average 1.6±SD = 1) in a single SP. The mean abundance was 3.1±SD = 2.3. (Table 3)

**Table 3:** The occurrence and abundance of IAPs in municipal urban woodlands. Calculated on the dataset of 1040 Sampling Plots (SPs).

Forest	The number of SPs	The number of SPs with IAP presence (%)	The number of IAP species	The share of SPs with IAPs (%)	Mean number of IAPs in all SPs	Mean abundance of IAPs in all SPs
Bemowo	170	121	13	71%	1.1	1.6
Bielany	63	55	10	87%	1.9	2.8
Bródno	75	57	13	76%	1.2	1.8
Kabacki	293	128	13	44%	0.5	0.7
Lindego	16	16	7	100%	3.6	6.9
Matki Mojej	31	24	10	77%	1.7	3.5
Młociny	71	37	8	52%	0.6	0.9
Na Kole	27	27	11	100%	3.4	7.4
Nowa Warszawa	16	15	4	94%	1.3	2.4
Olszynka Grochowska	53	47	10	89%	1.4	2.0
Sobieskiego	223	129	10	58%	0.8	1.2
Wydma Żerańska	2	2	3	100%	2.0	5.5
Total	1040	658	15	63%	1.0	1.6

### RF models of IAP abundance of frequent and rare species

**Fig 5:**
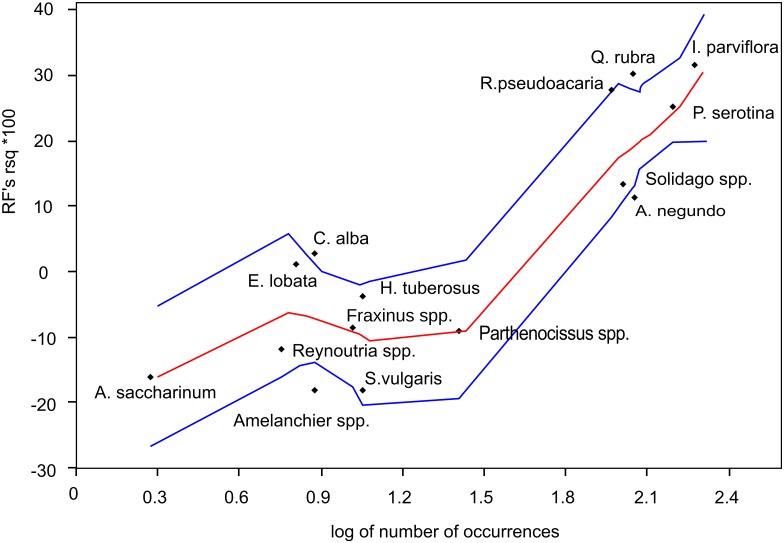
The RF model quality as a function of the number of occurrences of species from frequent and rare species groups, per 1040 SPs. Model quality is expressed as an RF's pseudo R-squared parameter, being an equivalent % of explained variance. Red line = LOWESS smoothing with smoothing coefficient = 0.5; blue lines = 2.5 and 97.5 percentile confidence intervals of LOWESS line.

RF models explained different portions of the IAP abundance variance. The best were models for frequent species, explaining from 25 to 33% of abundance variance (*Impatiens parviflora*—32.3%, *Quercus rubra*—30.3%, *Robinia pseudoacacia*—27.6%, *Prunus serotina*—25.1%), while the abundance of two frequent species was explained from 10–20% (*Solidago* spp.,—13.2%, *Acer negundo*—11.2%). All rare species' abundance was explained in much smaller part, and their pseudo R-squared was equal to or less than zero (Fig 5).

The quality of all models is strongly correlated with the log number of the presence of species in sampling plots. The Spearman correlation coefficient has high value (r = 0.74), and is highly significant (at p < 0.001).

There is a pseudo R-squared variability in both frequent and rare species groups. The abundance of *R*. *pseudoacacia*, *Q*. *rubra*, and *P*. *serotina* among a frequent species group and the abundance of *E*. *lobata* and *Cornus* spp. in a rare species group have been explained in a larger part than other species, especially *A*. *negundo* and *Solidago* spp., as well as *Parthenocissus* spp., *H*. *tuberosus* and *Reunoutria* spp.

The LOWESS trend line has two different parts for rare and frequent taxa. For rare species (log N < 1.5) the slope of the line sections is almost horizontal, while for more frequent species (log N >> 1.5) the slope is much steeper. Four data points lie behind the confidence interval lines: *Amelanchier spicata* and *A*. *negundo* are below the 2.5 percentile, and *Q*. *rubra* and *Cornus* spp. are over the 97.5 percentile. Moreover, the width of confidence intervals for rare species is almost twice as wide as for frequent ones. Thus there is a large decrease of average values and slight increase of variability of model quality with decreasing species frequency.

### The importance of predictors

Twenty-three of 56 predictors were found ‘Confirmed’ by Boruta algorithms for frequent taxa, while only 9 for rare taxa. The Total Sum of IncNodePurity Importance (ΣΣINP) differed even more greatly—it was 218 for frequent taxa and only 20 for rare ones.

Predictors fall into three broad categories according to their significance and importance. The first group can be called ‘strong and universal’ predictors—confirmed in at least half of all models or in at least half of frequent taxa and in at least a single rare one. They have ΣΣINP from 29.4 to 79.3 (average 54.3). Here the single most important predictor is FOREST NAME, with ΣΣINP = 79.3, with 6 models confirmed for frequent taxa and 5 for rare ones (79.3, 6, 5), followed by 16 distance predictors describing land use (e.g., distance to agriculture and waters (63, 6, 2), distance to river ports (59.2, 5, 6), distance to dense urban areas (58.5, 6, 2) and many others), three distance predictors from seed sources (with distance to seed sources of *R*. *pseudacacia* (63.3, 6 and 1), distance to seed sources of *Q*. *rubra* (42.5, 3 and 1) and distance to seed sources of *A*. *negundo* (42.0, 4 and 5)), and two tree stand predictors (real vegetation (113, 8, 1) and distance to forest patch border (46.2, 5, 1)). The second group can be called 'facultative and specialization' predictors—confirmed in at least one model. They have ΣΣINP from 2.8 to 48.5 (average 17,9). Here are mainly predictors describing forest properties (e.g., distance to city centre (38.8, 3, 0), forest area (6.6, 1, 1), urban infrastructure abundance (2,8; 2, 0)), tree stand properties (e.g. tree species dominating in canopy (34.4, 3, 0), vegetation deformation level (24.5, 2, 0), stand age (24.2, 1, 1)) and facilitative presence of other IAPs (e.g., abundance of *P*. *serotina* in lower layer of canopy (22.5, 1, 0), abundance of *A*. *negundo* in lower layer of canopy (16.4, 1, 0), abundance of herbal IAP (3.6, 0, 1)). The rest of predictors were named ‘marginal’. That group embraced predictors found ‘Confirmed’ for any model. They have ΣΣINP from 2.0 to 7.7 (average 4.5). Most of them are facilitation predictors (e.g. abundance of *Solidago* spp. (6.0), abundance of *R*. *pseudacacia* in understorey, (5.9), abundance of *A*. *negundo* in understorey, (5.1)). There are also some soil (e.g. soil fertility (4.7), soil cover shade tolerance index (4.0)) and stand predictors (canopy density (7.7), vegetation deformation (5.8), stand naturalness index (5.0)). The importance of those predictors is practically as low as ‘shadow’ predictors, obtained during randomization in the Boruta procedure. (S4 Table)

The values of all mean ΣΣINP and confirmed status for all Boruta models can be found in S5 Table (for frequent species) and S6 Table (for rare species).

### Summary of variable group importance

**Fig 6:**
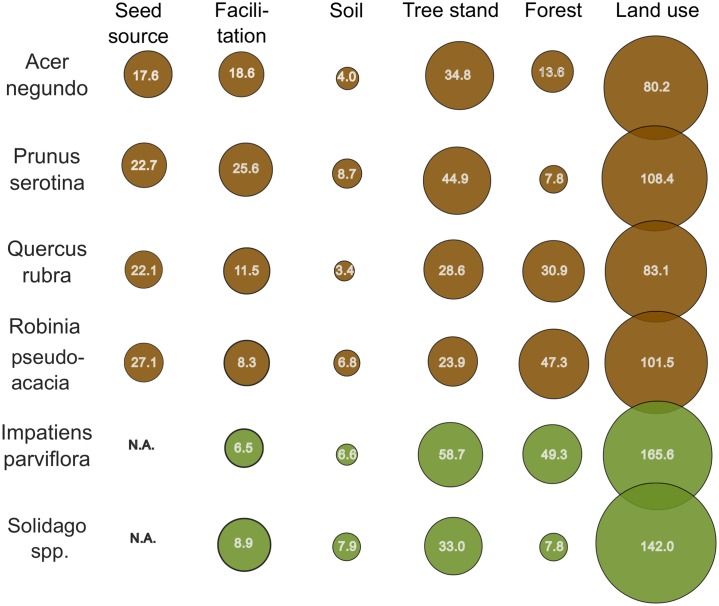
The sum of RF importance for variable groups for frequent species. The numbers in circles are the subtotal of RF IncNodePurity importance based on the sum of squared residuals. The area of circles is proportional to the share of a given group in the sum of importance for the model of each species separately. Brown colour indicates woody and green indicates herbaceous species; N.A. = data not available.

**Fig 7:**
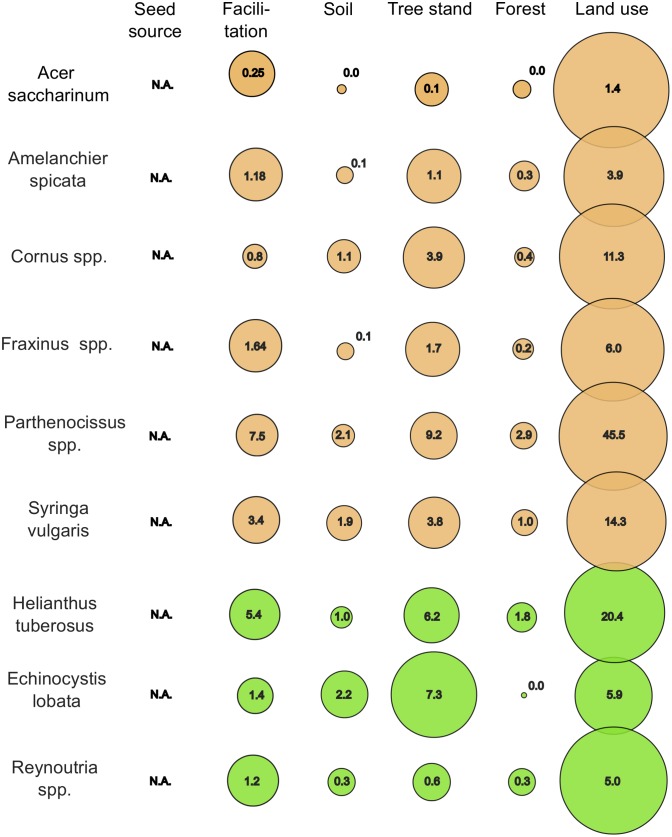
The sum of RF importance for variable groups for rare species. The numbers in circles are the subtotal of RF IncNodePurity importance based on the sum of squared residuals. The area of circles is proportional to the share of a given group in the sum of importance for the model of each species separately. Brown colour indicates woody and green indicates herbaceous species; N.A. = data not available.

The most important predictor group for all species was land use variables (Figs 6 and 7). The sum of IncNodePurity RF importance scores (680.8) for land use predictors across all models is higher than other predictor groups together. The second predictor group is tree stand properties, with the sum of IncNodePurity RF (224.2) less than one third of land use. Even less important groups are forest properties (157.0) and facilitation predictors (142.6), and the least important ones are seed source (68.5) and soil predictors (37.7).

The domination of land use predictors is stronger in herbaceous species—in the model of *Solidago* spp. land use predictors are responsible for almost two thirds of the whole sum of importance (142.0 of 219.2) and in *I*. *parviflora* more than half of the sum (165.6 of 312.4). The share of land use predictors in woody species is slightly lower, ranging from 46% in *Q*. *rubra* to 50% in *P*. *serotina*. The share of tree stand predictors varies from around 20–60% in all of the six frequent species, with the lowest in *R*. *pseudoacacia* (23.9) and highest in *I*. *parviflora* (58.7). The share of forest properties varies strongly, from less than 10% in *P*. *serotina* and *Solidago* spp., to almost 50% in *R*. *pseudoacacia* and *I*. *parviflora*. Facilitation predictors in four species vary at around 10% and only in *P*. *serotina* reach over 25% and in *A*. *negundo* almost 20%. Seed source predictors in woody species vary between 17% in *A*. *negundo* and 27% in *R*. *pseudoacacia*. Soil predictors vary between 3% and 9% with the lowest value in *Q*. *rubra* and highest in *P*. *serotina*.

The sum of IncNodePurity for rare species (187.7) is less than one seventh the sum for frequent species (1311.0). Despite this disproportion, for rare species land use is again the most important group of predictors (Fig 6), and the sum of IncNodePurity (114.2) for land use is again equal to the importance of all other predictor groups together. The next group is tree stand properties, with the sum of IncNodePurity (34.3) reaching less than one third of the value for land use, followed by facilitation (22.8), soils (9.2) and forest properties (6,9). The seed source predictors have no IncNodePurity values, as there were no data available.

There are no radical differences between the rank of importance of predictor groups for herbaceous and woody species. Only *Helianthus tuberosus* has an exceptionally high value of importance of tree stand and soil predictors, while *Parthenocissus* spp. does for land use.

The share of land use predictors varies generally between 1.5% and 20.5%, only in *Parthenocissus* spp. reaching 45.5%. The share of tree stand properties varies generally between 6.7% and 22.1%, and only in *Helianthus* reaches 43.5%. The mean share of facilitation predictors varies at around 13%, with only *Cornus* spp. and *Parthenocissu*s spp. remaining below 10%. The mean share of soil predictors is below 5%, except for *Helianthus* reaching 13.5%. The mean share of forest properties predictors is 3.4% and does not exceed 6% in any species.

### Hierarchy of predictor average importance for frequent and rare species

The direct analysis of covariability reveals that the relative importance of variables from groups is highly consistent across whole frequency range. The slope of all curves is more steep for low-frequency species than for high-frequency ones.

**Fig 8:**
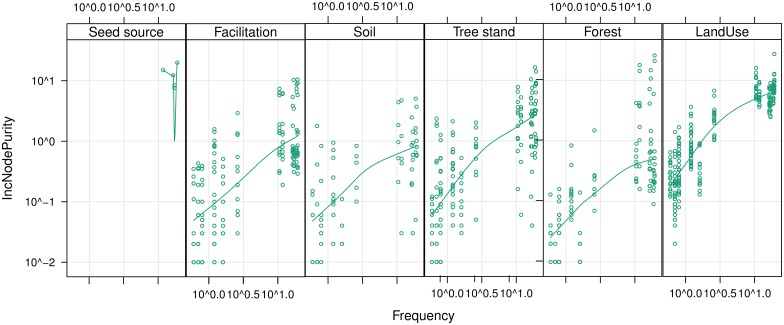
The dependence of predictors’ IncNodePurity on the frequency of species of concern, for different groups of predictors.

The series of points placed vertically in the plot, representing always the same set of variables, have considerable variability in both frequency groups. Therefore, the positive correlation cannot be easily attributed to a simple statistical effect of growing sample size (Fig 8). It is possible that variables’ importance represents the third curve shape from Fig 1, named explanation stagnation.

**Fig 9:**
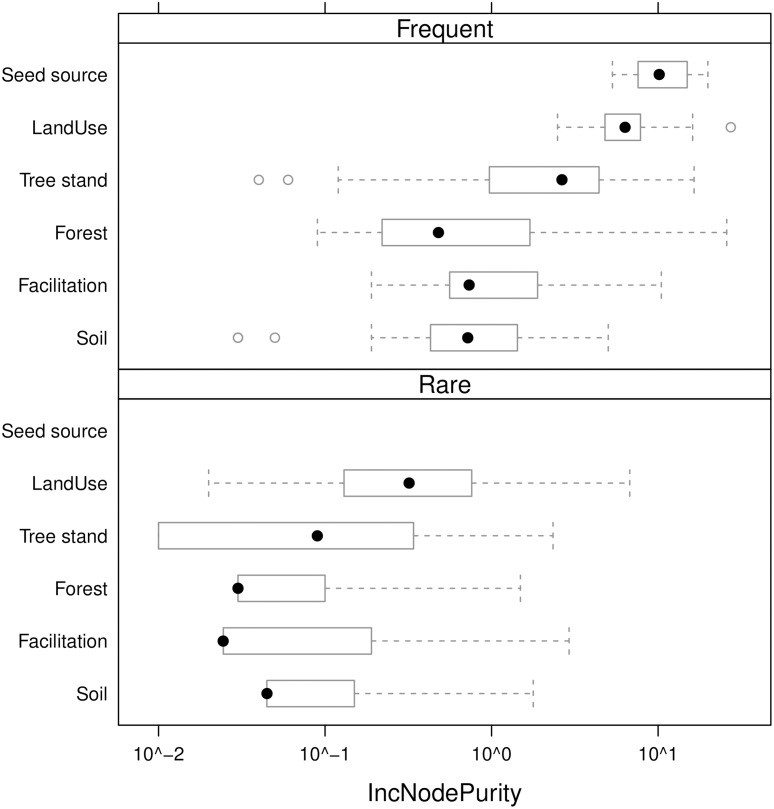
The variability of IncNodePurity values of predictors belonging to different groups for rare and frequent species. Full dots = medians, boxes = the first and the third quartile of variability (IQR), dashed lines = IQR*1.5, and empty dots = outliers.

The rank of importance of variables from different groups is similar in both rare and frequent species groups. The least important are predictors belonging to the Soil, Facilitation and Tree stand groups; Forest ones are of medium importance and the Land use is most important. Seed source variables are the most important single variables, however they were assessed directly only for a few frequent species. This confirms that rare species undergo a similar, though not identical, set of limiting and fostering environmental factors (Fig 9).

**Fig 10:**
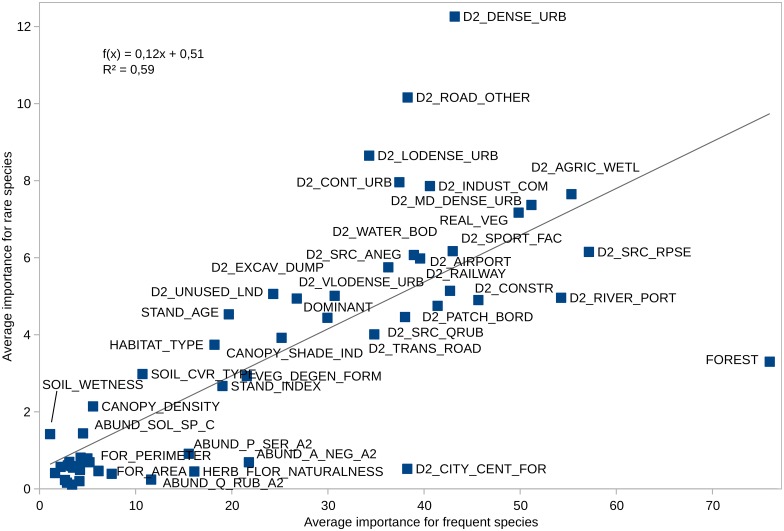
The average importance of single predictors for frequent and rare species groups.

Most single predictors are eight times more important for frequent than for rare species. However, there are predictors with comparatively higher importance for rare species than for frequent ones (Fig 10). They include: distance to the dense urban fabric, distance to other roads, distance to low dense urban fabric, distance to continuous urban fabric, and distance to industrial and commercial areas. There are also predictors with comparatively lower importance for rare species than for frequent ones: forest complex properties, distance to river ports, distance to the centre of the city, abundance of *Prunus setrotina* in the lower canopy layer, herb layer naturalness, and the abundance of *Q*. *rubra* in the lower canopy layer.

**Fig 11:**
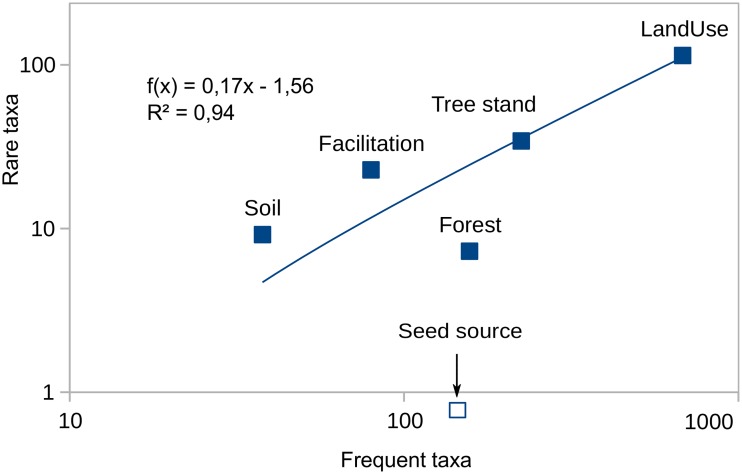
Sum of importance of groups of predictors for frequent and rare species.

As for the predictor groups, the sum of importance values for frequent species is seven times greater than for rare ones, which correlates with differences in model quality. The hierarchy of importance is roughly the same for both groups, while the group of soil predictors is more important for rare species, and properties of forests are comparatively more important for frequent species. The place of seed source predictors is ambiguous, because the sum of their importance has the fourth-highest score for frequent taxa and there have been no predictors from this group tested for rare taxa (Fig 11).

## Discussion

### Model quality of IAP taxa abundance

Most of our RF models are far from perfect. The best of them explained ca. 30% of local abundance variance, which is similar to the R^2^ values obtained in other studies (Table 1). The remaining 70% should probably be attributed to factors acting in other spatial scales, especially the scale of microhabitats, and the specificity of IAP regeneration niches [67]. Other groups of determinants that are possible, but missing in our models, may be interspecific interactions, perhaps assessed on the single individual level. In the end, it is also possible that IAP abundance—which we adopted as a response variable in our models—is in large portion stochastic, especially among rare species; so it is unreasonable to expect that their behaviour would be explained through static models with general predictors.

Such stochasticity seems to be a logical explanation of the shape of the LOWESS curve (Fig 5) for rare and frequent species. For frequent taxa, the explained variance strongly grows with increasing frequency, but for those with lower frequency there is no such change. This would then mean that: (1) the increase in model quality may not be a simple statistical effect, as it does not occur for all taxa and all frequency values; (2) the stochasticity may be smaller among frequent taxa; and (3) the most probable biological effect may be an ‘explanation jump’ when passing along the axis of rare-frequent taxa (Fig 1).

Perhaps other statistical explanations should also be considered, e.g. sensitivity of different algorithms to low values of sample size. Wisz et al. [68] considered response curves of many possible algorithms to the sample size variation, including lack of sensitivity and the sensitivity over the whole range of sample size as well as sensitivity to the particular low sample size. They found that while some modern algorithms similar to Random Forest, (like GBM) were sensitive to the small sample size, no algorithm was able to predict consistently well when sample size was small, i.e. below 30. This and other studies [69,70] found that Maximum Entropy is another reliable algorithm good at modelling with low sample sizes. However, in a direct comparison by Williams et al. [71] Random Forest performed even slightly better than Maximum Entropy at predicting new occurrences of rare plant species, seemingly based on a low number of occurrence points.

The question arises: do these results preclude the sensibility of modelling rare IAP species? We do not think so. We agree with Wisz et al. [68] that the use of predictions based on low sample size should be very conservative and restricted to exploratory modelling. We do, however, find it even more risky to try to infer ecological explanation through oversampling or other mere changes in sampling strategies where the original frequency of presence cases is very low and the data imbalance very high. If the species occurs scarcely and does not saturate the biological space, the analysis of its distribution cannot tell the whole story about its requirements and preferences, because its preliminary distribution is determined more by random seed spread than environment selection. Artificial oversampling of these few cases in the dataset would result in considering them the norm, which they are not. Thus, the results of the modelling of rare species using Random Forest should be used with caution even for early explanatory purposes. This also highlights the importance of gathering of new occurrence data in early stages of IAP invasions, as each new point may increase the quality of models and the precision of quantification of a species’ potential niche.

### The hierarchy of predictors for frequent and rare species

Besides general lower absolute values of predictors, frequent and rare species had surprisingly similar patterns of the importance of predictor groups (Fig 11). The hierarchy of importance is led in both groups by land use, then tree stand properties, forest, seed source, IAP facilitation, and soil. Frequent and rare species differ in low-grade, relatively weak factors, among which there are nevertheless quite a few relevant ones. Most evident is the difference for forest predictors caused by the single strong predictor FOREST NAME, embodying most unique local properties of each woodland, which are much more important for frequent species than for rare ones. Soil and facilitation predictors are comparatively more important for rare species, which results from the higher relative importance of habitat type, stand age, or soil cover, as well as distances to the areas with IAPs in the tree stand. But even the widely important land use predictor group (Fig 11) has two separate groups of variables: most of them are slightly more comparatively important for rare species, but the distances to various forms of urban fabrics and non-transit roads are much more important for rare species than for frequent ones. For them, three land use parameters are comparatively more important: the distance to fast transit roads, construction sites, and river ports.

Both predictor group hierarchies—for frequent and for rare species—are different from our expectations (Seed source > Soil > Tree stand >> other ones) based on similar earlier studies. For example, for the IAP density in the coastal upland near New York [72], most important were abiotic factors, then biotic interactions, with land use predictors in third place, followed by economic factors, photosynthesis intensity, and precipitation. For IAP taxa density on coastal sand dunes in central Italy [73]), seed source was definitely the most important predictor, followed by the habitat and soil-dependent factors. In other larger-scale studies, e.g. in Catalonia [74], the most important group of predictors for IAP species richness were climatic ones, followed by habitat diversity and land use as a third group. There are indeed studies in which land use predictors emerged as the most important ones: e.g., IAP species’ richness and spatial distribution in New England counties’ forests were mostly determined by the land use predictors, namely housing density and interface type with the forest, accompanied by forest connectivity, plants productivity, and rainfall [75]; in a large-scale analysis of IAP numbers in European countries and islands, human population density and wealth were decidedly the most important determinants [76].

While there are always possibilities that the determinants of any process may differ in various scales of analysis [6], the most probable explanation is that land use can be both a direct determinant of IAP invasion—affecting dispersal, survival, growth, or reproduction of IAP individuals—and an indirect determinant, masking the real ones [77]. Land use can act on the IAP invasion also in other ways [78], e.g. by increasing or decreasing air and soil pollution or by local modification of climatic conditions that can foster certain species, e.g. *Ailanthus altissima* [79]. However, the most probable predictor is the seed source. As the seed source data are missing in many models—especially of rare taxa—they may have caused an increase of the importance of land use predictors, indirectly related to the presence of seed sources [78].

There may be a real mechanism of these relationships, e.g. there are often rich sources of seeds located in ornamental plantings, e.g. in residential gardens (*Parthenocissus* spp.) and in street plantings (*A*. *negundo*). All of those mentioned above, as well as species that are also cultivated in the forest (e.g. *Q*. *rubra*), or have not been cultivated recently (e.g. *Solidago* spp.) or were never cultivated (*I*. *parviflora*) may often form large self-regenerated populations in less intensively managed areas. This in turn is strictly determined by the patterns of land use outside the forest and may contribute to the overall importance of land use predictors in our models. This explanation is additionally justified by the high per-variable importance of seed source variables for woody species. Where available, they were almost as important for single species as land use variables. Even if seed source variables inside the forest were available for a given species and included, there can always be additional seed sources outside the forest that fostered IAP invasions alongside inside-the-forest sources. The explicit analysis of this relationship would require other methodologies of study, including symmetrical research in both inside- and outside-the-forest environments [80]. However, one cannot forget that the inclusion of land use / land cover in IAP models has advantages, which is that they do not necessarily have to be direct determinants to be good predictors. Predictive functions of species distribution models, while not explored in this paper, are practically as important as regressive ones [48].

### Do frequent and rare species represent the same process of colonization?

The rising importance of ‘seed source’ in this and in other research on IAPs recalls the long-running dispute over whether ecological communities are niche assemblies, approached through complicated niche-based models based, e.g., on heterogeneity both in space [81] and time [82], or dispersal assemblies, as explained by simplistic, dispersal-dependent neutral models [83] and the unified neutral theory of biodiversity and biogeography (UNTBB) of Hubbell [84]. The interim conclusion of the latter dispute was that both driving forces, niche and dispersal, were important, and it is clearly the relative abundance—i.e. the frequency—which make the species sensitive or not to these determinants. According to UNTBB, niche dimensions or separation are more important for frequent taxa, usually with superior competitive abilities over the rest of the community. Rare taxa need not be competitively weaker, but due to their rarity they do not face pressure to find their unique niche in order to survive; they simply rely on their good-enough adaptations but are sensitive to dispersal limitations that hamper recolonization and the average frequency of the species in a ‘metacommunity’. This seems to be the case for IAPs in Warsaw’s urban forests as well. For the IAP taxa, a metacommunity with its ‘propagule rain’ can consist of an inside-the-forest population of planted woody of IAPs and outside-the-forest IAP population, whether it be spontaneous (e.g. *I*. *parviflora*), or cultivated (e.g. *A*. *saccharinum*), or both (e.g. *A*. *negundo*). The average frequency of a species in a metacommunity is clearly determined by land use patterns, and especially by the way taxa are selected and planted [85].

Obviously, an outer source of seeds would be more important in earlier phases of invasion due to both populational and spatial reasons. In the presence of an abundant seed source, the role of initial seed production by an escaping population is relatively small. For the abundant population in an advanced phase of colonization, the outer seed source—while still abundant—is no longer an only option, and its local importance may be dependent on the local advance in the process [86]. According to the UNTBB theory, more frequent taxa should be more averse to competitive neighbourhoods, which is also the case for Warsaw’s urban woodlands.

## Conclusions

1. The quality of RF models is strongly positively correlated with the frequency for frequent taxa, and shows no correlation for rare ones. It seems that along with increasing frequency there is an explanatory enhancement resulting from the increasing number and the constancy of species-environment relationships.
2. Predictors for frequent taxa are on average eight times more important than for rare ones. The most important predictor group for both rare and frequent taxa is land use, followed by the tree stand, facilitation and whole forest properties. Frequent taxa’s abundance depends more on the forest properties and distance to the seeds sources, as well as the distance to the city centre. Rare taxa’s abundance depends more on the distance to the different forms of residential areas and minor roads. This result can be interpreted as an indirect estimation of the importance of seed-source.
3. Contrary to our expectations, rare taxa appeared to be more dissimilar in their response curves to predictors. This may result from more random occurrences near the seed sources. Frequent taxa—colonizing forests much more commonly—more often occur together. This result is a warning against mechanical application of oversampling of rare classes in cases of imbalanced data, which can enhance model quality without removing the main weakness of data: scarcity of information on the species preferences.
4. Random Forest models are well suited to be applied to IAP taxa modelling, even to taxa of relatively low density—keeping in mind that the lower the frequency, the more sparse the environmental sampling is for the species, and the higher the random variation of modelling results—as even RF is not a crystal ball.

## Supporting Information

S1 Table Characteristics of studied forests. Columns contain most common values for the forests within locations marked on Fig 2.
(XLSX)

S2 Table Characteristics of studied taxa. (XLSX)

S3 Table Characteristics of all variables used in the analysis. (XLSX)

S4 Table The average of IncNodePurity RF importance and the number of Boruta ‘Confirmed’ decisions for frequent and rare species. (XLSX)

S5 Table The average of IncNodePurity RF importance and the number of Boruta ‘Confirmed’ decisions for frequent species. (XLSX)

S6 Table The average of IncNodePurity RF importance and the number of Boruta ‘Confirmed’ decisions for rare species. (XLSX)

## Abbreviations

IAP: invasive alien plant
RF: Random Forest (software)
SP: sampling plot
